# “Involved in something (*involucrado en algo*)”: Denial and stigmatization in Mexico’s “war on drugs”

**DOI:** 10.1111/1468-4446.12761

**Published:** 2020-06-08

**Authors:** Claire Moon, Javier Treviño-Rangel

**Affiliations:** 1 London School of Economics and Political Science, London, UK; 2 Independent human rights researcher, Mexico

**Keywords:** bystanders, denial, Mexico, stigmatization, victims, war on drugs

## Abstract

This article responds empirically to the question posed by Stan Cohen about “why, when faced by knowledge of others’ suffering and pain—particularly the suffering and pain resulting from what are called ‘human rights violations’—does ‘reaction’ so often take the form of denial, avoidance, passivity, indifference, rationalisation or collusion?”. Our context is Mexico's “war on drugs.” Since 2006 this “war” has claimed the lives of around 240,000 Mexican citizens and disappeared around 60,000 others. Perpetrators include organized criminal gangs and state security services. Violence is pervasive and widely reported. Most people are at risk. Our study is based on qualitative interviews and focus groups involving 68 “ordinary Mexicans” living in five different Mexican cities which have varying levels of violence. It investigates participant proximity to the victims and the psychological defense mechanisms they deploy to cope with proximity to the violence. We found that 62 of our participants knew, directly or indirectly, one or more people who had been affected. We also found one dominant rationalization (defense mechanism) for the violence: that the victims were “involved in something” (drugs or organized crime) and therefore “deserved their fate.” This echoes prevailing state discourses about the violence. We argue that the discourse of “involved” is a discourse of denial that plays three prominent roles in a highly violent society in which almost no-one is immune: it masks state violence, stigmatizes the victims, and sanctions bystander passivity. As such, we show how official and individual denial converge, live, and reproduce, and play a powerful role in the perpetuation of violence.

We all know from experience how the human tendency to self-delusion likes to declare dangers null and void even when we sense in our hearts that they are real.

Stefan Zweig, Beware of Pity

Knowing what not to know becomes not only an art of survival but the basis of social reality.

Michael Taussig, Law in a Lawless Land: Diary of a “Limpieza” in Colombia

The only logic is that of acquiescence.

Joan Didion, Salvador

In the terse opening paragraph of *If this Is a Man*, Primo Levi recalls life under Italy's racial laws. He describes how, in the years preceding his arrest by fascist militia in December 1943, he lived “in an unrealistic world… a world inhabited by civilized Cartesian phantoms” (1987, p. 19). These phantoms—faith in reason and its power to deliver civility—were so powerful that on his arrest, Levi immediately declared himself Jewish in the tragic belief that it would save him from the certain torture and death that would follow, instead, any disclosure of his anti-fascist activities. Levi was promptly taken to a transit camp near Modena where around 650 others were also detained. The arrival of a German SS squad did nothing to arouse their suspicions: “we still managed to interpret the novelty in various ways without drawing the most obvious conclusions… despite everything, the announcement of the deportation caught us all unawares” (p. 20). The group was beaten while being forced onto buses that took them to the Auschwitz-bound train. “We received the first blows and it was so new and senseless that we felt no pain… only a profound amazement” (p. 22). The group then boarded one of the “notorious transport trains, those which never return, and of which, shuddering and always a little incredulous, we had so often heard speak.”

“Unaware,” “amazed,” “incredulous.” These words shed light on the necessary self-deception that protected Levi and his companions from the horror that awaited them at the end of their journey. Only after travelling for five grueling days, arriving at the camp, and being stripped, shaved, and tattooed did the grip of denial loosen. “We are tired of being amazed… we seem to be watching some mad play” (p. 31).

Earlier in the century this phenomenon had been labeled “repression” by Sigmund Freud (1954 [1900]) but subsequently assumed the more popular nomenclature of “denial.” It is this latter which concerns us here. Differently from Freud we are concerned with *denial as a social phenomenon* in mass atrocity contexts and are guided by Stan Cohen's seminal work on denial and human rights (2001). Following Cohen we ask “how ordinary, even good people, will not react appropriately to knowledge of the terrible. Why, when faced by knowledge of others’ suffering and pain—particularly the suffering and pain resulting from what are called ‘human rights violations’—does ‘reaction’ so often take the form of denial, avoidance, passivity, indifference, rationalisation or collusion?” (1993). However, where Cohen is concerned with the passive consumers of information about “distant suffering” (Boltanski, 1999), we instead animate this question in relation to proximate—and often intimate—bystanders to atrocity and suffering *who are also at risk*. Our context is Mexico's so-called “war on drugs,” about which news and other media regularly circulate accounts and images of executions, mass killings, and newly discovered mass graves, including images of corpses and dismembered bodies deliberately discarded in public places. What is more, international and national human rights organizations all produce high profile and widely publicized reports on human rights abuses perpetrated by the police and military against civilians including torture, extra-judicial killings, enforced disappearance, and opportunistic violence. Perhaps most significantly, however, many Mexicans have experience of kidnappings, disappearances, violent detention, torture, and killings because their family members, neighbors, colleagues, and other intimates have been directly affected. This complexifies bystander agency because many are likely vicariously traumatized by their proximity to violence, and as a result of the often indiscriminate and random nature of both criminal and state violence, coupled with high numbers of victims, are also vulnerable to victimization themselves.

With so much information available and experience of the violence so widespread, no-one in Mexico can claim not to know. And yet, there is clear evidence of political and social denial of the violence. Our task is to understand the nature of denial in this context, and to show how political and social denial collude to generate and perpetuate a powerful narrative about “who is to blame.” Our study involved qualitative interviews and focus groups involving 68 “ordinary Mexicans” living in five different Mexican cities of varying levels of violence. We wanted to investigate participant proximity to the victims and to explore their psychological defenses. We found that 62 of our participants knew, directly or indirectly, one or more (and up to seven) people who had been illegally detained, kidnapped, killed, or disappeared. We also found that their psychological defense mechanisms—primarily victim-blaming—echoed prevailing state discourses about the violence. One dominant rationalization for the violence was produced and reproduced by the state and society more broadly: that the victims were “involved in something” (drugs or organized crime) and therefore “deserved” their fate. We argue that this discourse of “involved” plays three prominent roles in a highly violent society in which no-one is immune. The first of these is political: justification for the state's “war on drugs.” The second and third are social: stigmatization of the victims and sanctioning of bystander passivity. In sum, we show how official and individual denial converge, live, and reproduce in a highly violent society.

## 1 Denial

Since Freud introduced the concept of denial (repression) to psychoanalysis it has become a catch-all term to refer to a range of psychological strategies for handling information—truths or emotions—too difficult to admit into consciousness. Denial is a psychological defense mechanism that facilitates the *selection of information* about one or another theme, issue, topic, feeling, idea, or memory over another. It “ensures that what is unacceptable to the conscious mind, and would if recalled arouse anxiety, is prevented from entering into it” (Davis, 2004, p. 803).

Denial has a rich life in popular discourse and a vocabulary all of its own. It is commonly expressed by sayings such as “burying your head in the sand,” “wearing blinkers,” “looking the other way,” or “turning a blind eye.” It also goes by the names of suppression, repression, avoidance, passivity, indifference, rationalization, minimization, collusion, and disavowal, amongst others. These are all “general and fairly nonspecific terms for matters that are left out of awareness in order to avoid the noxious emotions specific to the personal significance of such awareness” (Edelstein, Nathanson, & Stone, 1989, p. ix). Denial can be parsed into three rough types: outright denial (the overt denial of a fact, emotion, or behaviour); minimization or rationalization (acknowledgment of the fact, emotion, or behavior but downplaying or rationalizing its significance); and projection or misattribution (attributing a fact, emotion or behavior to someone or something else).

### 1.1 Denial as negative

Edelstein notes that “despite the attitude of scientific objectivity” that characterized Freud's original work on denial “it has since taken on a negative connotation, and those who use this avoidance system are seen as the lesser among us” (Edelstein et al., 1989, p. ix). This negative interpretation is, with good reason, dominant in contemporary work on the relationship between denial, state crimes, and human rights (Cohen, 1993, 1996, 2001; Cohen & Seu, 2002; Seu, 2003, 2010, 2013). This body of work is most salient to our discussion because it concentrates on two phenomena central to human rights and their violation—one political, one social. The first is denial by the state of crimes such as torture and genocide. The second is bystander passivity towards violence and suffering.^1^

Cohen's distinctive and powerful typology of denial—literal denial, interpretive denial, and implicatory denial (2001, pp. 7–9)—has been central to advancing a sociological analysis of both state denial and bystander passivity. In Cohen's scheme, literal denial refers to the type of denial in which the fact or knowledge of the fact is denied outright, for example “X was not tortured”; “there was no massacre”; “nothing happened here.” By contrast, interpretive denial does not refute the basic facts but imputes a different meaning to them, for example: “it's not really torture, just ‘enhanced interrogation’”; “it's not ethnic cleansing but ‘population transfer’”; “the victims were not really victims because they were ‘involved in something’.” In interpretive denial “officials do not claim that ‘nothing happened’, but what happened is not what you think it is, not what it looks like, not what you call it” (2001, p. 7). It is characterized by minimization and passivity. The third type—implicatory denial—does not attempt to deny the facts nor their usual interpretation. Instead it denies, rationalizes, or minimizes the moral, psychological, or politi*cal implications*. For example, “the suspect is a ticking bomb and torture is justified under the special circumstances [insert Cold War, war on terror, war on Iraq, or whatever ongoing concern] in which we find ourselves”.^2^

Each of these types of denial have their own, internally varied, psychological status. Literal denial might be a result of genuine ignorance, blatant lying, a psychological defense against an intolerable truth, or a “cultural not-noticing because the reality is part of your taken-for-granted view of the world” (Cohen, 2001, p. 9). Interpretive denial might be “a genuine inability to grasp what the facts mean to others,” or a cynical re-description to avoid moral responsibility or legal accountability. Implicatory denial often draws upon popular explanations (“banal folk techniques”) “invoked with mystifying degrees of sincerity” to avoid moral or psychological censure (2001, p. 9), such as “it's always gone on… what can you do about it?”

While Freud's intra-psychic model of denial is the building block on which Cohen and Seu develop their theses, they use it as a window onto their discussion of denial as a political and social phenomenon. For Cohen, the main objects of analysis are “public, collective and highly organized” denials that are structured and made possible by the massive resources of the state (1995). For Cohen, “denial is… not a personal matter, but is built into the ideological facade of the state” (2001, p. 10). The recent denial by senior Myanmar officials of the genocide against Rohingya Muslims comes to mind (see Beech & Nang, 2018). By contrast, Seu (2003, 2010, 2013) examines broader social or cultural denial through the example of public reactions (namely passivity) towards human rights appeals such as those by human rights organisations such as Amnesty International. She investigates the ways in which social or cultural denial is manifest in a range of everyday “folk tales” that are invoked to neutralize moral claims: “giving money's like putting a little bandage on a big wound”; “it's just the same thing, over and over”; “what difference can I possibly make?” Cohen argues that there is a “mutual dependency” between these two phenomena, between “official lying” and “cultural evasion” (2001, p. 11). In our case, this is reflected in the way in which the Mexican government's narrative of “involved in something” has permeated popular discourse, as we will show, and has become a routine political and popular explanation for hundreds of thousands of deaths and disappearances in Mexico.

Cohen’s and Seu's work on denial and human rights is especially interesting, we think, if read as an extension of Becker's (1973) influential work on death denial.^3^ Becker argues that bloody ideological conflicts—wars against communism, terror and so on—are the inevitable outcome of “immortality projects,” which are defense mechanisms arising out of “our” (modern, Western) fear of death. According to Becker, we attempt to transcend death by giving ourselves over to grand schemes—moral, ideological, religious, or otherwise—through which we participate in something we believe to be of great and lasting worth and for which we are prepared to shed blood *en masse*. The connection with Cohen and Seu lies in an irony. Where Becker's denial of death *engenders* war, genocide, torture, and the rest, Cohen’s and Seu's denial can be read as a coping mechanism that both alleviates the discomfort of proximity to violence, suffering, and death *and* avoids responsibility for it. On this account, denial appears as the *constant* companion of violence, both contributing to its appearance *and* its continuation, as we will discuss.

### 1.2 Denial as positive

Despite the dominance of the negative view of denial, Edelstein refuses to attach a judgment to it, wishing, rather, to preserve the “scientific objectivity” of Freud's original idea. In this spirit, he argues that “the mechanism of denial is not immutably pathological but may be used in the service of psychological health” (Edelstein et al., 1989, p. 173). The word originally used by Freud to refer to “defence mechanism” is synonymous with “shield,” thus denoting the psychic strategies that protect humans from reality. This is what we mean by “positive denial” insofar as denial actively serves positive psychological ends in contexts where there are few options but to re-describe what is happening, even if, as we argue later, the social and political consequences of this re-description are negative. On this interpretation, denial can be seen as a “skill and a defence” insofar as “what is denied cannot be solved and is best left unanswered” (1989, p. x).^4^ Seu (2016) describes this as “adaptive denial,” which is employed when no alternative course of action is available. This is sometimes expressed as an *active* turning away. Some of our respondents demonstrated this. Leticia said “I pretend that this is no longer happening… I used to watch or listen to the news, but only the minimal details… because I don't want to hear about beheadings. That disturbs me.” Maria-Angel responded that “I don't look for news about violence in the papers… I avoid it. It doesn't give me anything positive to be reading this stuff. Absolutely nothing.” These quotes show our participants trying to continue to function in a deeply disturbing reality of widespread and seemingly random violence.

This positive interpretation is not fully acknowledged in prevailing analyses of denial and human rights—with which we are in dialogue—and we aim to explore and address this in our analysis. We pitch our discussion between these negative and positive perspectives in order to argue that social denial accrues complicated cultural significance in the context of societies in which atrocities are widespread, ongoing, and in which bystanders are at risk. While our analysis contributes to thinking about bystander passivity as a significant negative consequence of denial, we also aim to extend a legacy of thinking about denial as a survival mechanism, originated by studies following the holocaust.

## 2 Horrorism and Terrorism: Mexico'S “War on Drugs”

Some context is necessary. The presiding narrative about recent violence in Mexico marks its beginning with the controversial 2006 presidential election which saw Felipe Calderón beat his opponent, Manuel Lopez Obrador, by less than a percentage point. The election was distinguished by irregularities, recounts, and widespread civil protest and came at the height of a summer of spectacular criminal violence particularly in the state of Michoacan, which has a long history of drug production and trafficking (Maldonado Aranda, 2013). One notorious episode in Uruapan, one of Michoacan's largest cities, saw five severed heads dumped onto a dancefloor along with a *narcomensaje* (threatening message) left by the killers. [Correction made on 30 June 2020, after first online publication: *‘narcomanta’* has been corrected to *‘narcomensaje’* in the preceding sentence] In another, seven corpses, shot in the head, were publicly displayed with *narcomensajes* pinned to their chests with ice picks. [Correction made on 30 June 2020, after first online publication: *‘narcomantas’* has been corrected to *‘narcomensajes’* in the preceding sentence] The men, thought to be windscreen washers who worked the busy roads, were each sat upright on seven of those generic white plastic garden chairs and placed on a roundabout at a busy junction in Uruapan. Like an impromptu and surreal theatrical performance. In another episode, a severed head was left next to an infant playground in Veracruz. And in another, a man denuded of arms and legs was discarded on a dirt road, laid out on his front with his head propped up on his chin, his hair still gelled, unnervingly, in place. Dancefloors, roundabouts, and playgrounds are not places one would normally expect to encounter a corpse, still less a dismembered one. Such “horrorism” (Caravero, 2011) is distinguished by massacre and disfiguration in which “the end melts away and the means become substance. More than terror, what stands out is horror” (p. 1). Horrorism is primarily deployed by criminal organizations to exact revenge and compete for power. It is endlessly creative in its barbarism and has become the distinctive feature of Mexico's “war.” Yet, there is another crucial but under-reported story to tell: that of state terror.

Shortly after his election and in spite of the fact that there had been no notable increase in criminal violence for 20 years (Escalante Gonzalbo, 2011), Calderón announced his “war on drugs”—which he dressed up as a moral appeal to the family to mourn and transform Mexico's “drug affected sons”—to underwrite his tenuous political legitimacy with a show of force.^5^ Calderón promptly commissioned Mexico's military to the streets along with large numbers of federal police to “combat drug trafficking” and “create an atmosphere of peace, security and social stability” (Sedena, 2012, p. 134). This “war” was to become Calderón's own immortality project and the insignia of his political leadership. It was to seal his authority, his political fame, define his premiership, and outlast it.

Since the launch of this “war”, the number of illegal detentions, torture, and killings carried out by the security services (military, navy, and police) under the guise of controlling drug trafficking, increased dramatically (Atuesta, 2017; Pérez Correa, 2015, p. 17; Treviño-Rangel, 2019). Military and police checkpoints now pockmark Mexico and are frequent sites of extortion and violence, witnessed by the mass graves that cluster around them (Paley, 2014, p. 110; author interview with forensics expert, 2015). The military and police perpetrate serious, widespread, and random violations of human rights to the extent that “torture and ill-treatment” have become “generalized in Mexico” according to Méndez, UN Special Rapporteur on torture (2014). The situation is compounded, *facilitated* rather, by the fact that official investigations into killings and disappearances are rare. Between December 2006 and January 2011 of 35,000 homicides attributed by the authorities to organized crime, only 22 led to criminal convictions (HRW, 2011, p. 15). *Twenty-two*. That is, 0.06%. That means, for practical purposes, 0% (Schedler, 2014, pp. 11–12). Put otherwise, impunity rates for that period run at 100%.

Calderon's “war” was, ostensibly, launched against criminal organizations with the aim of controlling a set of illegal economies including but not restricted to drugs, such as the illegal extraction of profit from transnational migration, human, sex, and organ trafficking, arms trafficking, the extractive industries, fuel siphoning, water appropriation and other natural resources, commercial agriculture and fishing, as well as an expanded business portfolio that includes investment in construction, tourism, restaurants, and the automotive industry. However, the government and its institutions is implicated in, and profits from, the exercise of many of these activities through what is a historic, almost century-long relationship between the state and organized crime (Astorga, 2005; Encisco, 2010). Some of the most recent evidence of this relationship emerged in December 2019 when Genaro García Luna, former minister of public security under Calderón and an architect of the “war on drugs,” was arrested in Texas on charges of protecting the drug-trafficking activities of El Chapo Guzman's Sinaloa cartel in exchange for millions of dollars of cash bribes. And iconic of Mexico's atrocities is the case of Ayotzinapa, in which 43 (still missing) students were abducted in 2014 in Iguala, Guerrero by police officers working with criminal gangs. It is well documented that the state works with criminal organizations and profits from their activities (Correa-Cabrera, 2018; Mastrogiovanni, 2014; Pansters, 2018; Pimentel, 2000; Treviño-Rangel, 2018), that criminals are protected by the state and act with impunity (Flores, 2014), and that some cartels have military origins, notably *Los Zetas* (Correa-Cabrera, 2017). Organized crime works with the police, public servants, and judges in one or more levels of government (municipal, state, federal) or all at once (Schmidt & Spector, 2015, p. 1). Members of the police and military work as or with *sicarios* (hired killers), or they protect criminal activities. Indeed, criminal activity cannot flourish without the complicity of, and protection by, the authorities (Schmidt & Spector, 2013). The fact that criminal organizations flaunt their crimes in public, confident of their immunity, is evidence of both tacit and active complicity by public servants and institutions. As such, the distinction between the state and organized crime is nonsensical in Mexico and, it follows, any alleged “war” by the state against organized crime.^6^ This enduring relationship makes the subjects (who is involved?) and objects (what is it about?) of this “war” obscure. Who or what is at war with who or what?

As a result, we avoid the regular term “organized crime” (*crimen organizado*) altogether. Much more apt and powerful, we think, is Schmidt and Spector's term “authorised crime” (*crimen autorizado*) (2013, 2015). This has the advantage of a phonetic similarity to the original with a clever subversion. It compresses everything you need to know about crime, violence, and impunity in Mexico in one pithy phrase.^7^

Calderon's war continued under the administration of his successor Peña Nieto (2012–2018) and continues today under that of Lopez Obrador (2018–present). Contrary to official claims it has not led to a reduction in violence but has killed almost 270,000 and disappeared around 60,000 people to date (Comisión Nacional de Búsqueda de Personas, 2020). Homicide statistics for 2019 stand at 34,582 (Secretariado Ejecutivo del Sistema Nacional de Seguridad Pública, 2019a, 2019b), the highest since records began. *The homicide increase is so significant that it has contributed to a reduction in the country's projected life expectancy* (Csete et al., 2016, p. 1433). Yet Calderon and Peña Nieto both denied state responsibility for the increase in homicide rates. They claimed that it was the result of confrontations between drug cartels and have persistently and consistently presented those detained and killed by security forces as “involved in something” (drugs and/or organized crime), and therefore “getting what they deserve.” This “cartel wars discourse” is distinguished by “an almost exclusive reliance on state and government sources for information, a guilty-until-proven-innocent and victims-were-involved-in-drug-trade-bias, and a foundation belief that cops involved in criminal activity are the exception not the rule, and that more policing improves security” (Paley, 2014, p. 35).

Under such conditions—widespread atrocities, indiscriminate violence, the involvement of organized criminals and the state in its perpetration, state denial and the failure of state institutions to address it—what option do “ordinary Mexicans” have other than to look away?

## 3 Researching Denial

Data were collected through semi-structured interviews and focus groups involving 68 Mexicans resident in Mexico.^8^ The decision to include focus groups was based on an intuition that a group setting might provide a more generative environment in which to speak given the sensitivity of the subject matter, and thus deepen the findings. This was sometimes confirmed where there was a mute response to a question until one person spoke, then others, previously silent, offered more information. Participants sometimes seemed more willing to talk about traumatic experiences when they felt that these were shared. Focus groups also occasionally facilitated memory prompts.

Residents of five cities—Aguascalientes, Mexico City, Guadalajara, Durango, and Torreón—were selected due to the varying levels of violence in those cities, listed here in order of least to most violent.^9^ We were concerned to generate a “general picture” rather than determine differences in responses according to more or less violent cities. Participants were recruited by snowball sampling which was appropriate to the sensitive nature of the data being collected. Recruitment was controlled to ensure that the group characteristics broadly corresponded with those of the population: it contained men and women aged 18–65, from different class backgrounds, geographic areas, and occupations. Participants were also selected on the basis of their “ordinariness”: they were not, to our knowledge, members of political parties, journalists, members of the security forces, nor members of criminal gangs, nor were they socially or politically involved in drugs war activism.^10^

Participants were asked whether they *knew directly* (a relative, friend, neighbor, colleague, or acquaintance), or *knew of* (someone known *to* a relative, friend, neighbor, colleague, or acquaintance) anyone who had been illegally detained, kidnapped, killed, or disappeared. They were asked about their perceptions of the violence (context and causes), their attitudes towards the victims, whether they had acted on information about violence (reported it to the authorities, for example), and whether they had taken any personal precautions (to determine whether they had *acted* to protect themselves). Participants were also asked about the responses of authorities to reported crimes, whether cases were investigated, and whether they thought that they or families of the victims could “do something” to change the current situation. They were also asked if they had engaged in any social protest about the situation.

We encountered a number of difficulties in researching denial, two of which stand out. First, in the process of asking questions it was difficult to avoid prompts, to which participants sometimes modified their responses. They might recall something forgotten, for example. As a result we became implicated in the “breaking” of denial and changed, however subtly, the thing we set out to study. This problem is intrinsic to all research to varying degrees, but became more “live” in the context of the subject of this study. Concomitantly, we potentially disturbed a crucial defence mechanism by probing it. Second, we risk appearing to criticize our research participants. However, our intention in this study is to consider denial as both positive and negative, and also to illustrate empirically Cohen's argument that “the rationalisations that governments produce are not mere rhetorical flourishes” but are nationally and culturally rooted (1996, p. 542).

## 4 Knowing the Victims

We found that most participants had *intimate familiarity* with the violence—62 out of 68 participants *knew directly or knew of* someone who had been illegally detained, kidnapped, disappeared, or killed since 2006.^11^

A number of participants reported arbitrary and illegal detentions by the military, police, and navy, many based on trumped-up charges or extortion. Some led to disappearances within the detention system itself. Laura said “I know many cases. The main one happened last year. A person went to jail but not for something wrong he did. It was a mistake… he is still there”. Libra reported, “I know not only one, but hundreds of cases and I know directly those affected, and I can see the impotence they feel because they can't do anything about it.” He went on “there were no motives. And every branch of the security forces—the municipal police, state police, federal police, the military and the navy—does this kind of thing.” Another said “a friend of mine had some dope on him. Just one spliff. Nothing else. It was less than 30g, which is legal for personal use here. But he was illegally detained by police officers who beat him up and planted a kilo of dope on him and he was taken away.” Others reported illegal detention with extortion: “my cousin was detained by members of the federal police who bribed him. ‘Look, you piece of shit, if you don't give us money we will…’ And he had to give them money.” A picture emerged of significant knowledge of arbitrary detentions by security forces. The victims were frequently illegally detained on the assumption of being “involved in something” or knowing someone who was. Illegal detentions appeared random, as did subsequent releases, where these took place.

Some reported kidnappings. Christian reported “at least seven people who have been kidnapped.” Another said:

My son was travelling from Culiacan… He had his young son with him. They went to eat tacos and he got out of his car to order them, but left the kid in the car. When he got back to the car, someone trained a gun on him and said “take the car away”. The gunman said “Don't pretend you're not who you are”. They threw him into the back of the car. Another guy got in the car and they were driving with my son and grandson for two hours around the city. The child was vomiting out of fear, and asking “father, what are they going to do to us?”. After two hours one of these guys called someone on a radio and my son could hear the person on the other end saying “Just kill him. Kill him off”. But the kidnappers were saying “he seems to be clean, he doesn't seem to be involved in drug trafficking and he has a child with him”.

Many reported knowing someone who had been disappeared. A disappearance is countable as such *if the person has not reappeared* either alive or dead.^12^ “Disappearance” can refer to any number of things, including detention by security personnel, kidnapping by criminal gangs for ransom, forced labour, or sex-trafficking. One participant spoke of a famous case in Aguascalientes: “They went to a nightclub and there were builders, workers and managers working there, and they took them all. They never reappeared.” Jesús said “my cousin is missing. He was living in Leon. He went missing in 2010, his wife pregnant, and we haven't heard anything from him. We don't know if he was killed, kidnapped or had an accident.” Laura spoke of “the uncle of my ex-boyfriend. He was picked up by the federal police… He's been missing for a year.” She added, “however, he was a former member of the federal police,” as though this explained the disappearance. Jessica did not seem to remember at first but then said, “actually, a father of a friend. Well, it wasn't a disappearance. It was a kidnapping. He came back alive.” Maria said “no, I don't know anyone. *Only my nephew*,” as if this did not warrant mention. She went on: “he was kidnapped in front of a school and the school had cameras so we know it was the police because they were wearing police uniforms.” Others reported variously that: “the father of a student of the French School was kidnapped. He was… never found. They don't know where he is. He was in his 80s and he was sick”; “Paco's son disappeared. Nothing is known about what happened. It seems he was mistaken—his brother was involved in bad stuff—but he was kidnapped and was never found.”

Many knew people who had been killed. Claudia said “he was taken away and… killed after two months. But a year went by, and the family was still being asked for money.” Paco reported that “the uncle of a very good friend of mine… was kidnapped and later his head and fingers were found in an icebox on the highway towards Zacatecas.” Diego said “yes… He wasn't really my friend, more like an acquaintance. He'd been selling drugs for a long time… he was shot in a bar along with his brother and another guy and they took their corpses and hanged them [in a public place].” Nina said “there was a party… all the people in the party were killed.” (Interviewer asks “ALL of them?”). She responded, “there were not too many. Just four or five,” as though it was an insignificant event. Alejandro said “yes, a distant relative of mine… his head was found somewhere.” Laura reported knowing five people who had been killed. Another said “the son of a couple I know. He… got involved with narcos. He made a lot of money. He moved to another city, maybe because of problems… he was disappeared when he was leaving a nightclub. He was found… with terrible signs of torture.”^13^

Many of the focus group participants knew someone who had been killed or were perhaps more willing to talk about it in a group setting: “a friend from junior high school. We all knew that he started using and then selling drugs, and so on. He was my neighbour and was killed a few years ago… His body was disposed of in front of his house. His mother crossed the street to get rid of the garbage and she found the body of her son there”; “my neighbour was found dead on the street outside his place”; “a friend of mine was working in a bar. He was picked up and left outside his house in little pieces”; “a young guy was disappeared. His family was looking for his remains, and I know that they found them”; “they were looking for the person until they found them dead. In the first case organised criminals did it. In the second case, I don't know. People even talk about a satanic sect because the murder was extremely brutal.” This last comment echoes the ways in which people construct folk tales—“satanic sects”—to explain what happened. Another said “I know one person killed by narcos, one professor of the school I studied at was kidnapped, then later he was found in little pieces in a garbage can”; “a friend was taken away whilst he was working in a bar, and he was found in little pieces outside his house.” Heads, fingers, little pieces. Many participants bore witness to horrorism. It underpinned a credible fear of unchecked violence.

## 5 Blaming the Victims

The most striking and sometimes visceral responses were directed towards the victims. Participants expressed a strong belief that victims were “involved in something.” This refrain came up time and again. Not coincidentally, it was also the key state justification for the vastly increased rates of disappearance and homicide since 2006. These increases, the governments of Calderón and Peña Nieto claimed, were the result of “criminals *killing each other*” (Presidencia de la República, 2010).^14^ Calderón repeatedly stated that this accounted for 90% of deaths.^15^ We found this justification (including, sometimes, this “statistic”) to have been internalized by our research participants and, by extension we argue, Mexican society more widely.^16^ At the same time there was a significant awareness that *anyone could be affected*. Many participants knew victims who had not been “involved” and seemed aware that both criminal organizations *and* state security forces were implicated. This contradictory state of awareness presents an important fissure, an instability in denial, to which we return in our concluding discussion.

### 5.1 Involved in something

Alicia stated that “victims are connected to narco-trafficking… I really don't believe that victims are civilians. They are directly in contact with the narcos and are involved in narco-trafficking. I don't see that a completely innocent person could be kidnapped.” Jessica said “I don't think oh, they've killed some saints. I think these people are involved in some kind of conflict. I don't think they're killed randomly… What I really think is… that… victims have done something.” Maria said “they must have been involved in *that* milieu in order for something to happen to them,” and Tonita that “almost all of them are involved in drugs. This is what happens to mafia members.” Monica argued that “victims are people who are within the same circles (as narcos). Always. Of course, they *had* to be involved in something… this is the way it is. This is what happens to the poor guy who was working as a dealer, and the guy working as an informant. These are the people who are killed.” Alejandro said “you realise that in general victims are involved. At the very least, they have a contact… So, in general, when you hear that so many people were killed, you always think well, it's because they were involved in something.” Another said “people… are killed because they are *sicarios* (hit men) or because they were in conflict with narcos.” Another, echoing the official narrative, that “90% of these people have some kind of relationship with narco-trafficking.” When asked about the discoveries of mass graves (500 bodies had been found in Torreón at the time of the study), participants said, variously: “perhaps they were bad guys”; “these people were involved in something”; “they were people who were involved in those things”; they “were involved with drug trafficking”; “victims… found in mass graves were involved with the narcos.” “Involved in something” was the dominant and crucial narrative that *both* stigmatized the victims *and* provided a psychological defence against the possibility of the same thing happening to them. As one participant put it: “if you aren't involved, there is no risk.”

### 5.2 They get what they deserve

Some participants were convinced that the victims got what they deserved. This “just deserts” narrative seemed more prevalent amongst the focus groups, which suggests that it was more strongly generated in a group rather than an individual setting. Miguel said “in the end he deserved what happened”; Paco that “all these people involved in bad things… deserve it. They know what they're exposing themselves to”; others that “this is just about revenge between criminals. People who are involved with cartels”; “… the fact that so many are being killed in such an ugly way is due to the kind of life they have chosen. *They get the death they choose*”; “this is more or less what we all think. When we talk about this issue… we say is that these people were behaving badly so had to be taught a harsh lesson”; “they deserve what they get… if they're linked to violent and brutal criminal gangs, they're exposing themselves… they are involved. It's going to happen”; “if criminals are fucking with people, they should be fucked.” “Getting what they deserve” is an important narrative in Mexico. It expresses an underlying belief—or more likely hope—that some kind of social order is being maintained, that there is justice even if it is “rough” (“criminals killing each other”). These examples of victim-blaming evince a “just world theory” (Lerner, 1980) or cognitive bias towards the idea that “bad” behavior is punished “in the end” and that “good” behavior is rewarded. Lerner suggests that this belief might be important for the maintenance of well-being insofar as it serves psychological defense. This is especially powerful when innocent people are thought to be suffering. In such cases, Lerner argues, events are rearranged in order to reinterpret the victim as deserving of their fate. Our research provides a powerful illustration of this theory.

### 5.3 Staying away from them

Some respondents said that it was better to stay away from victims. Participants were asked “what would you think or do if you heard of a neighbour disappearing?” One said “it'd depend if the victim was involved in something.” Another added: “I would think badly of him.” A third said “I would take care myself, because this person was definitely involved in something so criminal activity could also affect me.” Paco put it like this: “my neighbours would gossip about it… my neighbours would probably avoid the relatives of the disappeared person… because we don't want to be seen as related to them. People think the same will happen to them if they don't distance themselves… .” A symbolic boundary, charged with stigma, is created. “They” (the victims) are different from “us,” they put us at risk and are best kept at arm's length. Victims and their families are thus polluted by the experience of “something happening to them.” “Staying away” has a social impact on the victims and their families, who frequently feel isolated and stigmatized by their communities.^17^

### 5.4 Not all victims are equal

Some respondents expressed ambivalence about the victims, saying that some were innocent, that no one is completely safe. However, an acknowledgement of innocence was often quickly followed by a shift of attention to those who were not, in the view of the speaker, or was preceded by an assumption of involvement. Lalo said “of course, anyone can be [a victim]. However… [for those] that are on the edges or involved, It's more likely that something will happen to them.” Another said “If I hear that the victims were families, or children or teenagers I think that's terrible, but if they're the victims of the settling of accounts amongst narcos then I think that's good because they were involved in something so that's the way they had to end.” Another said “when I think about victims I can't avoid reaching the conclusion, first, that they were doing something wrong… second, I think, well, who knows? There have been cases of many people that were in the wrong place at the wrong time, that they were confused, and you know, criminals never forgive. But the first thing I think is that they were doing bad things.” Another agreed: “I think exactly the same.”

Sometimes participants expressed helplessness or “feeling bad” for victims who were not involved. For Jessica, “if something happened It's maybe because the victim was involved in something. Maybe not. But anyway, you… can't go and rescue them, can you?” Miguel said “I feel bad because I think It's unfair that these people are paying for the problems of other people.” These views were sometimes differently inflected depending on the proximity of the victim to the speaker. One participant said “we take this (that they were involved) for granted. If It's someone who is not close to you, you can't think otherwise.”

### 5.5 Awareness: Everyone is at risk

The dominant perception that victims were “involved in something” was often accompanied by the conflicting perception that anyone was at risk. Participants said: “ordinary people tend to be victims because those in power are untouchable”; “you can no longer label those who get killed 'as something' (that is, as a cartel member)”; “*anyone* can be killed or disappeared”: “I think we are all potential victims, but I think you have to *carry on doing your stuff*.” “Carrying on regardless” sometimes accompanied this acknowledgement of vulnerability. It was an expression of helplessness.

“Everyone is at risk” conflicts strongly with the idea that victims are “involved in something.” It seemed rooted in the high level of belief that the security forces—police and army—were killing innocent people. Participants said: “when I see these heavily armed trucks, with these assholes on the top of the trucks with their machine guns you think… these beasts will accidentally start a stupid shoot out if they drive on a bumpy road! Pulp fiction! Far from making us feel secure, they scare us!”; “I think It's stupid that security forces are on the street because the only thing they do is to… provoke deaths. There are many reports… even about children who are shot because they didn't stop at a checkpoint”; “I'm scared of the police. When I pass in front of them or when a police van drives next to me I feel very anxious.” Yet others implied that the police were involved with cartels. One said “those who are Zetas are clearly recognised by the police. And they are never touched. I think there's some kind of agreement between Zetas and the police.”

These simultaneous states of awareness—that victims are criminals and that the army and police are also killing innocent people—create a psychological conflict that produces discomfort and anxiety. They are also evidence that denial is precarious rather than stable. The attempt to stabilize the narrative results in the stigmatization of the victims and the attempt to secure the self through the general (and widely shared) idea summarized by one participant's comment that “if I'm not involved in illegal stuff, if I'm not doing anything outside of the norm, if I live a normal life, if I don't mess with specific people, nothing should happen to me.”

## 6 “Denialism” and “Denial Proper”

The political discourse that the victims are “involved in something,” inaugurated by Calderón and perpetuated by Pena Nieto, is a powerful example of denial. It does not entail *literal* denial (the first of Cohen's three types): the state acknowledges that people have been killed, tortured, and disappeared. However, it does entail *interpretive* denial, the second of Cohen's types, insofar as officials and bystanders claim that “what happened is not what you think it is” (2001, p. 7). As such, the moral significance of state violence is *minimized* by the suggestion that those killed were involved in criminal activities and were therefore not “really” victims. The discourse of “involved” is also an example of *implicatory* denial, the third of Cohen's types. Implicatory denial rationalizes the political and social consequences: the victims were criminals and *therefore got what they deserved*. It provides a *rationale* for their deaths and, by extension, for the “war on drugs” and the militarization of public security. It is effectively a “denial of the victim” (Cohen, 1996, p. 531). Interpretive and implicatory denial are characterized, respectively, by minimization and rationalization. They are abundant in the Mexican political and social discourse on the “war on drugs.”

We argue that two types of denial in Mexico might be distinguished theoretically as “denialism” and “denial proper.” Denialism is a position that systematically refuses the facts for ideological purposes (Nelken, 2016, pp. 453–454). This describes the state discourse of “involved” that underpins the “war.” But further than that, “Denialism is Denial writ large—when an entire segment of society, often struggling with the trauma of change, turns away from reality in favour of a more comfortable lie” (Specter cited in Nelken, 2016, p. 456). Denialism thus captures the ways in which political and social denial are interconnected and made more powerful by virtue of that interconnection. By contrast “denial proper” might be understood as the psychological defence mechanisms that ordinary Mexicans deploy to distance themselves from the violence. It is mechanically distinct from “denialism” insofar as it has no *ideological* impetus, but is driven by *anxiety and fear*. However, denial proper can *look like* denialism insofar as it draws on and reproduces political ideologies (comfortable lies) such as “involved.”

## 7 Cartesian Phantoms *Redux*

The discourse of “involved” has accrued such popular authority that it now masquerades as common sense and plays two prominent roles in a highly violent society in which no one is immune. The first of these is political: justification for the “war on drugs.” The second is social: stigmatization of the victims. It has become the unrivalled “cartesian phantom” (rationalization) that stigmatizes the victims, sanctions bystander passivity, and masks state violence. Let us look at these consequences in more detail.

First, the discourse of “involved” generates a symbolic boundary that distinguishes those who are “involved” from those who are not. As Epstein argues, symbolic boundaries manifest “collective agreements about certain connotations that are… persistent” (1992, p. 236). These are “particularly acute during times of social change and upheaval” when “distinction blares out… as a trumpet call to arms… and becomes institutionalized in the patterns and practices of our lives” (p. 232). The political and social stakes in making and maintaining these boundaries are especially high in Mexico. Its political genesis is traceable to Calderón's inauguration, as we have shown. However, we have also shown that it has assumed a diffuse social life insofar as it also provides a social distinction between those who are “involved” and those who are not, which feeds into *both* the political justification for militarization (“they get what they deserve”) *and also* the popular rationale for the increase in homicides (“just criminals killing each other”). We have shown how state and individual denial converges, how it lives and reproduces, how it shapes both perceptions of the victims (they are “involved”) *and* behaviors towards them: killing them (the state) or staying away (bystanders). Its repeated articulation plays a role in producing and shaping political action and reinforcing the language and habits of everyday life. We have also suggested that this symbolic boundary has arisen out of the mechanisms of social control both at the level of the state—which has the power to shape a specific social reality that is advantageous to it and disadvantageous to others—and to the extent that it shapes social attitudes and behavior. Our data illustrate empirically Cohen's argument that there is a “mutual dependency” between “official lying” and “cultural evasion” (2001, p. 11). This has resulted in material consequences. The victim category now designates a social boundary, an *objectified* form of social difference, insofar as it is manifest in unequal access to resources such as justice.^18^ Evidence of this is implied in the impunity statistics cited earlier in our discussion, which, arguably, are material manifestation of the discourse that the victims “got what they deserved.” We might infer from these statistics that reported disappearances and killings are not considered to merit full and proper investigation. Habits of thought such as “classifying and demarcating” (Veblen, 1979 [1899]) are central to the mechanisms that produce boundaries between groups, particularly where the stakes in maintaining the distinction are high. They are central to the production of a moral distinction between those who “get what they deserve” and those who are unaffected by violence.^19^ Victim-blaming is thus bound up in the elaboration of a moral order which structures and regulates perceptions within a community (Durkheim, 1965 [1912]).

Second, victim-blaming is connected to a fear of pollution by the victims. This, we suspect, might have been behind the tendency of many of our participants to at first “forget” that they knew someone affected and only “remember” when prompted or when another member of the group volunteered that they knew someone affected. Victim-blaming is related to a fear of “being next” and thus attempts to psychologically secure bystanders from risk, albeit in a “phantasmic” way to use Levi's phrase. As a result we argue that the discourse of “involved” is a “pollution belief” that arises out of the impulse to impose order on (or “purify”) that which is inherently “untidy” (Douglas, 1966, p. 5). The untidiness of the classification “involved” was sometimes acknowledged by participants, and presented a threat to psychological security. They said that some victims *might* be innocent, that *anyone* might, inadvertently, be targeted, and they expressed a lack of trust in the security forces to target the “right people.” Such ambivalence causes “cognitive discomfort” (Douglas, 1966, p. XI). This was clear in some of the responses where an expression of ambivalence about the victims would be quickly followed by a re-assertion of blame. Matias said “whether they were involved or not, nobody deserves this kind of death. However, generally, you think that these people must have been involved, it was revenge, the criminals were after them.” This statement provides an insight into the attempt to assuage psychological discomfort, without which the speaker might be overwhelmed by anxiety and terror. Blaming sometimes varied according to the proximity of the speaker to the victim. The “involvement” of those close to the participant (such as a relative) was sometimes questioned. Angel said “my nephew was killed on the highway along with another young guy who was travelling with him… he was unlucky in being picked up. Some people are unlucky. They are sold or taken to *Los Zetas*. He wasn't involved in illicit activities.” Perhaps, after all, they simply happened to be in the wrong place at the wrong time. More distant victims, such as neighbours, were perhaps involved in something and it was considered preferable to stay away from them. Distance of association sometimes seemed more closely correlated with stigmatization.

Third, “involved” has both positive and negative dimensions. On the one hand, it operates as a psychological protection against a reality too terrible to contemplate and about which people can do very little. One said “if you've already been to the authorities and nothing has happened what else can you do?” Many reported feeling that they could do nothing to change the situation. Some spoke of their experience of trying to report a violation, of their exasperation, worry that the report was going nowhere, and fear of persisting. As Edelstein et al. argue, “in such situations, continued attention to pain and distress renders the organism dysfunctional; where denial can be used to foster a focus on the possible… then denial is a healthy mechanism” (1989, p. 2). We argue that this, positive, aspect of denial is not fully acknowledged in work on denial and human rights. For instance, Bauman argues that bystanders always play a “reprehensible role in the evil act” (2003, p. 137) and are on a continuum with perpetrators (see also Staub, 1989). This, he argues, is because they are always in danger of taking on the perpetrator's ideology and rhetoric. Certainly, we have provided evidence to confirm this in the case of Mexico. The psychological defenses of bystanders have incorporated and reproduce state propaganda. At the same time it is difficult to determine whether the bystanders are gullible dupes (although unlikely, since many demonstrated awareness of state complicity in atrocities), whether they are identifying with the state (some were), or whether they are simply drawing on the state narrative as a way of functioning under deeply threatening circumstances. The narrative of “involved” plays an immediate and plausible role in the management of anxiety and feelings of terror. Taking these things into account, we argue that denial should also be understood as an expression of vulnerability, and want to present a case for more nuanced and empathetic consideration of bystanders than Bauman's claim would allow. Such an approach might be recovered from early work such as that by Latané and Darley (1970), originators of the “bystander” term, who identify a particular stage of assessment (being able to help/make a difference) as a key step in the decision-making process leading to acting or not. Their work helps to make sense of why our participants, who clearly feel overwhelmed and helpless, fail to act and instead reach for the prevailing explanations that justify their inaction and disavow the victims. To this we would add that denial may also neutralize action that bystanders might also take *to protect themselves* since they believe themselves to be immune from risk if they are not “involved,” as our participants suggested. These positive and negative attributes of denial taken together create what we call the “denial paradox.” This paradox was evidenced by an important incidental finding of our research about forced displacement in Mexico. Some victims were reported as protecting themselves by moving away from their home towns.^20^ By contrast, bystanders were not taking such action. This finding illustrates the denial paradox at work: denial might provide psychological protection, but simultaneously expose those in denial to risk because belief in immunity from violence prevents bystanders from taking potentially self-protective action, such as moving to a less violence-affected town or city.

Fourth, we found that denial is *unstable*. Bystanders hold contradictory attitudes towards the victims (they are “involved”/“potentially innocent”) and towards the military (they are “killing criminals”/“killing innocent people”). This instability is supported by social boundary theory: no social category is internally consistent. As Epstein puts it “often the contents of the category are so unclear that it exists largely in terms of its symbolic boundaries” (Epstein, 1992, p. 236). This is particularly the case in Mexico where the distinction between “involved” and “not involved” is one that is so complex and socially embedded all the way from the street to the senate, with young kids acting as *halcones* (look-outs) for cartels in bus stations and taxi ranks, and politicians taking dirty money to influence political campaigns. [Correction made on 30 June 2020, after first online publication: *‘alcones’* has been corrected to *‘halcones’* in the preceding sentence] This instability is perhaps the most salient and positive finding with respect to thinking through the possibilities for changing the discourse, because while the distinction between victims and bystanders might appear to restrict social change, its “muddiness” may also permit it (Epstein, 1992, p. 236). On this account it might be possible to conceive of bystanders in Mexico as on a continuum with victims (differently from Bauman's conceptualization of them as on a continuum with perpetrators) insofar as many are traumatized by proximity to violence and also highly likely to become victims themselves. The possibility of bystanders becoming victims, and their consciousness of the precariousness of the distinction between them, might provide a source of challenge to the prevailing discourse. At the same time, the proximity of bystanders to victims might be a source of reinstating the distinction with more vigor: the higher the potential for victimization, the greater the need to instate psychologically the distinction from victims. That is to say, on the basis of our analysis we would speculate that the instability of denial in the Mexican context at least, in which violence is extreme and widespread, could make it susceptible to challenge *or* deeper entrenchment. In, again complex, illustration of this, Obrador's administration has taken some measures to change the official narrative about victims. It created the *Programa Nacional de Búsqueda y Localización* early in 2019, which seeks to find the more than 40,000 disappeared people in Mexico, investigate around 1,000 clandestine graves, and identify some 26,000 dead bodies that have been recovered by officials. This move has so far assisted the public visibility of victims and the legitimacy of their cause, and promises to challenge the established narrative. At the same time, state denial has proven to be highly adaptive. The drugs war discourse—originated by Calderón as a response to the specific political conditions of his election—allowed the government to benefit from organized crime while appearing to be “doing something” about it. Crucially this paradox persists today, albeit differently, under Obrador who campaigned in 2018 on the promise of investigating disappearances, human rights abuses committed by security forces, and advancing migrant rights. While Obrador appeared committed to human rights he simultaneously increased military funding and even added a new wing to it, the controversial new *Guardia Nacional* (see Corcoran, 2019; Meyer, 2019), which has taken on federal policing functions. Obrador has effectively further entrenched the militarization of public security, which is likely to lead to *more violations* of human rights, while appearing simultaneously to be *addressing violations* of human rights. Obrador also launched a new campaign against drugs that *stigmatizes drugs users by linking them to violent crime*. The campaign—*Juntos por la Paz* (together for peace)—attempts to unite state and society against the “real” enemy: drugs users. This program also lends legitimacy to the extension of military powers via the new Internal Security Law which “goes against Mexico's international obligations, as it perpetuates the military's presence in the streets and assigns them [sic] security tasks that are incompatible with the nature of their institutional mandate.”^21^ This potentially contributes to a new wave of victim denunciation. So, while as Solnit puts it, “key to the work of changing the world is changing the story,” it is clear that this is a complex and fraught process in Mexico and does not have a linear trajectory. In fact, Obrador's policies show denial's ability to renew itself.

Finally, “involved” has become *the* mode of classification “natural” to the “war,” the *par excellence* linguistic category that has been used to describe, launch, and justify violent political action. It has simultaneously made bystanders complicit with the crimes. As such, denial has become deeply implicated in the preservation of the dominant political order. The discourse of “involved” designates the victims as criminals and in so doing, scaffolds the state's distinction between violence perpetrated by the state versus that by criminal gangs and justifies the “war on drugs.” A final question to ask, then, is what system is being preserved by denial? And what is at stake in its preservation? In response to the former, we have illustrated the ways in which denial supports and perpetuates political ideology by providing a justification for state violence, and structures social order through stigmatization. These findings are plausibly extendable to other violent contexts in which the state is committing atrocities, criminal violence is widespread, and the general population is at risk, such as in Columbia or El Salvador to name a couple of examples. What is at stake in its preservation in Mexico is concealment of the deep enmeshment of the state with organized crime even though the veil on this relationship is occasionally lifted, such as in the recent trial in the US of El Chapo Guzman which saw claim that Peña Nieto accepted a $100 million bribe from El Chapo after taking office in 2012 in return for dropping the “manhunt” for him. Despite evidence, the drugs war discourse attempts to conceal the fact that the state and cartels co-profit from a relationship in which bribes are accepted by state officials in return for various protections, and, more significantly, profit from the operation of host of illegal economies including but not restricted to drugs, such as migration, land appropriation, the plunder of natural resources and so on. In other words, it disguises a de facto state of “authorized crime” which is central to mass killings and disappearances and in so doing, provides an artful concealment of the fact that the Mexican state is, effectively, at war with itself.

In sum, we have provided an empirical illustration of the ways in which drugs war discourses “provide an efficient smokescreen,” provoke “moral panic in the population,” and “calcify and exaggerate divisions among communities” (Paley, 2014, p. 19). We have also argued that “involved in something” has become the “vital lie” (Goleman, 1985) that enables the state to commit atrocities with impunity while “ordinary Mexicans” look away. It is the discourse most urgently in need of challenge.

## Data Availability

Data cannot be made available due to the security-sensitive nature of the research.

## Footnotes

1
**Endnotes**
This body of work has special relevance for our discussion because it focuses on political and social denial, although it separates the two. We advance this work by showing in our study how these phenomena are conjoined. Other works on denial of sociological note include Zerubavel (2006), who examines conspiracies of silence around the everyday (alcoholism) and around large-scale historical events (genocide). Additionally, Chancer and Andrews (2014) attempt to reunite sociology and psychoanalysis in order to facilitate thinking about the relationships between *psychic repression and social oppression*. In so doing, they aim to enrich sociology by renewing an interrupted conversation with psychoanalysis from which sociology had been, the authors argue, “unhappily divorced.”

2 Note that this justification acquired vigorous life in the post-9/11 context, favored by neo-cons such as Alan Dershowitz, and liberal intellectuals such as Michael Ignatieff alike.

3 The origins of the death denial thesis also lie in the origins of psychoanalysis. Freud wrote that we “have shown an unmistakable tendency to put death aside, to eliminate it from life… We cannot, indeed, imagine our own death; whenever we try to do so we find that we survive ourselves as spectators… every one of us is convinced of his immortality” (1918). Note that Becker's thesis is not without controversy and opposition. See Lofland (1978) and Kellehear (1984) for sociological critiques.

4 Seu's account of adaptive denial also includes an active turning away when alternative actions *are* available—an “I could but I won't” theory of denial.

5 The widespread narrative that Mexico is at war with organised crime emerged in 2006 and was fostered by the Calderón administration. This is the dominant narrative in most studies of violence in Mexico: 2006 is seen as the 'year zero' from which time the 'war on drugs' unfolded and violence increased exponentially. We do not concur with this narrative about violence and crime in Mexico. The perpetration of state crimes against Mexican citizens is historic and not contemporary, and the involvement of state agents in criminal activities can be traced to the beginning of the 20th century. This is not a story of change, but of continuity.

6 Notwithstanding Tilly's general argument that state-making and war-making qualify “as our largest examples of organised crime” (1985, p. 169).

7 It also presents an alternative paradigm to those of the “absent” or “failed” state, both of which have been used, inadequately, to describe Mexico.

8 The interviews and focus groups were carried out in 2012–2013 and should be read against the period of the “drugs war” from 2006 to 2013. Since then, the “war” has persisted and homicide rates increased and our findings can plausibly be extended to the present.

9 At the time when the interviews were conducted Durango and Torreon were highly violent cities (50 homicides per 100,000 inhabitants). Guadalajara was a city with medium levels of violence (20 homicides per 100,000 inhabitants). Mexico City had medium to low levels (12 homicides per 100,000 inhabitants). Aguascalientes was a city with low violence (4 homicides per 100,000 inhabitants). See Inegi (2018).

10 Participants’ names have been changed to ensure anonymity.

11 This calculation must be treated with caution as research methods were not designed to deliver precise quantitative data. Our methods of recruitment mean that we cannot rule out people reporting knowledge of the same incident. At the same time, a good degree of mitigation of this is provided by the fact that participants were not all associated and resided in five disparate cities.

12 Disappearances are not countable as killings unless remains have been found and identified, so some reports of disappearances may also be killings.

13 Participants would sometimes state that victims were narcos or “involved.” Yet when asked “how do you know that your brother/friend/neighbour/niece was involved?” it turned out that they had been informed of this by the police who often said things like “your relative disappeared because he was involved in something so stop with your enquiries or the same may happen to you.”

14 Contradictorily, there is evidence of an increase in disappearances and killings in places where state security forces are deployed (Atuesta, 2017).

15 “We have a serious problem with violence that results from the confrontation between criminal groups… last year, according to our investigations, based on the profiles of these people, on the activities they carried out, more than 90% of those who died are people who have been involved in one group or another, or distributed or transported drugs” (Calderón, 2009).

16 Note that it was not possible within the parameters of our research to establish which discourse came first, the political or the social, since our data on social denial were collected in the wake of the announcement of the “war” by the state. As such, we focus on the ways in which the political and social discourses within the period under research were mutually sustaining.

17 Ongoing but as yet unpublished research by Moon with the families of victims appears to confirm this.

18 We draw here on Lamont and Molnar's distinction between a symbolic and a social boundary where “social boundaries” are “objectified forms of differences manifested in unequal access to an unequal distribution of resources… and opportunities” (2002, p. 168).

19 While Veblen theorized distinctions between economic groups his insights are nonetheless relevant here.

20 Indeed, Mexico has a largely unacknowledged yet significant forced displacement problem on account of the violence (CONAPO y UNFPA, 2019; Papadovassilakis, 2019). Note that the CONAPO y UNFPA (2019) report cited here attributes forced displacement primarily to violence caused by criminal organizations rather than by the state. We would query this since the general levels of violence have massively increased since the state launched its “war” in 2006.

21 See https://www.wola.org/2018/11/mexico-supreme-court-internal-security-law/
